# Factors Influencing the Severity of Syphilis

**Published:** 1901-10

**Authors:** Bernard Wolff

**Affiliations:** Atlanta, Ga.


### FACTORS INFLUENCING THE SEVERITY OF SYPHILIS.

BY BERNARD WOLFF, M.D.,

ATLANTA, GA.

So long as the definite cause of syphilis remains unknown we are forced to confine ourselves to considerations that are in the main hypothetical and speculative. Its mode of acquirement, clinical course and reaction to therapeutic measures give strong presumption of the germ theory of its origin, although, as is well known, the proposition has not as yet been proven. The real genesis of the disease, chemical or bacterial, desirable as would be the accurate scientific knowledge of it, is in a measure only accessory to the subject in hand, for in any instance the same phenomena are presented to our attention, and the same problems have to be solved, whether empirical or scientific.

We are consulted by a young man, healthy and vigorous, who seeks advice concerning an eruption which proves on examination to be specific in character. The form of mercurial treatment deemed most suitable to the case is selected and under its influence there is a rapid and, so far as we are able to judge from long observation of the patient, a permanent disappearance of the symptoms of the disease. But the proposition is not always so simple. Our assistance is sought by another and equally healthy and robust young man, suffering from the characteristic lesions of the florid stage of syphilis. As in the first case, the proper treatment is instituted, but instead of prompt removal of the evidences of the disease, the lesions are stubborn and persistently recurrent. There are squamous syphilides, of the palms and soles, mucous patches of the tongue and throat, with perhaps the precocious appearance of the accidents associated with the later period of the disease. Despite the most judicious treatment, faithfully and intelligentlly carried out, the disease is more and more difficult to master, until at length it dawns upon us that we are confronted with an instance of malignancy in syphilis which defies the antagonistic action of drugs. What now is the distinction between the cases, the one mild and submissive, the other severe and rebellious? Does it reside in the quality of the infecting agent or the nature of the soil which receives it? This is a question which has never been satisfactorily settled, and one on which syphilographers take opposite positions, one faction being partizans of the theory of the seed, the other that of the soil. Both sides advance arguments and adduce proofs that are apparently irrefutable, so that the different elements of the problem are at wide disagreement.

It may be said that a majority of veneriologists adhere to the notion that syphilis is solely influenced by the nature of the soil in the one who contaminates and the one who is contaminated. The quality of the soil is influenced by the state of health, of development and the coefficient of resistance to disease of the organism, the presence or absence of diathetic vices, alcoholism, tuberculosis, malaria, gout, and by the race, temperament, age, sex, and occupation of the individual. In syphilis, as well as in other dyscrasiæ, very severe cases may be acquired from very benign ones, and *vice versa*, and this would seem to be the strongest argument in favor of the theory of the soil. That the infecting virus had fallen upon soil that was highly antagonistic to it and inhospitable to it, and though the virus retained its virulence unaltered, it was quite unable to cope with the self-protection offered by the healthy organism, and that under such adverse circumstances the infection lost its efficiency and became weak and inactive and readily put to flight by mercury, with the powerful cooperation of the *vis medicatrix naturæ.* According to this hypothesis the severity of syphilis and the intensity of the infection are directly controlled by outside influences, the source of the infecting material being in no way related to the behavior of the disease after transmission.

It is contended by opponents of this theory that the factors which influence the severity of syphilis, alcoholism, gout, tuberculosis, in a word, anything that weakens the organism and diminishes its power of resistance, apply with equal force to any other disease of similar gravity, and that they are empirical, banal and prove nothing. They further maintain that the specific effect of mercury over the malignant types of the disease is as great over the benign, and that, therefore, the disease retains its autonomy without prejudice to coexisting diseases. The partizans of the notion of the seed being the determining factor of the severity and intensity of the infection assert that its virulence resides in the disease itself. It is mild or severe in itself, and the quality of benignity is solely a matter of treatment. Their views succinctly expressed are as follows: The severity of syphilis is occasioned not by the nature of the soil, but by that of the virus. Syphilis contracted from a source which has not for unknown generations been subjected to treatment and consequently not attenuated, gives a syphilis that is severe and often fatal.*

Syphilis contracted from a source that has been attenuated by treatment, that is, that has been held in check by constant specific medication, remains latent or produces no manifestations, or at least very mild ones, and then only when the treatment is suspended.

Syphilis contracted from a methodically attenuated source, that is treated for a long time with mercury, is benign, and will recover rapidly under relatively small doses of the specific.

These aphorisms are illustrated by appropriate clinical cases.

There can be no doubt but that syphilis is much milder now than at the time of the siege of Naples in the fifteenth century, when it

*Gémy, quoted by G. Marcou, in *L'Indépendence Medicale*, to whom credit is given for much herein contained.

is said that the returned soldiers of Columbus began the dissemination of the disease throughout Europe, which was indigenous to America. It was pestilential and caused the death of many individuals. Yet we cannot say how much alteration syphilis has undergone during the centuries which have elapsed. I cannot help but think that the severity of the disease at that time was exaggerated, or that it was confounded with some more fatal and rapidly spreading epidemic of another disease. Syphilis is a disease of sexual origin, and is regularly acquired from contact during intercourse. If the Neapolitan epidemic were as fatal as has been represented, it is difficult to understand the ability and competence for sexual connection of the sufferer, in whom the loathsome appearance would be sufficient to frighten away from touching him any one short of St. Elizabeth of Hungary. Nor is the gloomy picture affirmed by the course of the disease among uncivilized or low grade peoples at the present time, in whom the physical condition as regards anterior experience with the disease is analogous to that of the inhabitants of Europe at the time of the return of Columbus. I may say in passing, that the embargo in some countries of Europe against certain American importations is, therefore, not without justification in an early distrust. The behavior of syphilis in virgin soil, according to the advocates of the theory of the seed, depends entirely upon the source from which it is derived, whether attenuated or not, and that its severity and intensity are governed by this condition and not otherwise. We have among us, it seems to me, a refutation of this doctrine and a highly valuable proof of the influence of the soil as modified by race upon the conduct of the disease. In the pure-blooded negro, in whom syphilis is exceedingly frequent, the disease runs so mild a course, in spite of incomplete treatment or no treatment at all, as to be practically self-limiting. The graver terminal lesions of the bones and viscera are rarely seen. This is a matter of common observation and is easily apparent to any one who has had an opportunity of doing clinical work among negroes. In marked contrast to the benign course of the disease in negroes, is the action of syphilis in the mixed type or mulatto. Among the latter the ravages of the disease are very great, not only as to the surface lesions, but also of those organs which are essential to life. As the source is practically the same in both instances, for mulattoes are classed with the negroes and live as one with them, the theory of the inherent virulence of the infecting material as the prime factor in the severity of the disease is seriously attacked.

It cannot be argued that syphilis is benign in the negro because its source is attenuated, and severe in the mulatto because it is not, for even though the source were common or identical, the intermingling of strains of relative virulence which must have occurred is enough to render the contention nugatory. The mild type in the full-blooded negro is constant in spite of his bad environment and notoriously vicious habits, and his pronounced proneness to tuberculosis. This fact speaks very strongly for an inherent resistance in the negro himself, otherwise we would logically expect to see the disease fluctuating directly with the quality of the infecting virus.

Having sketched the arguments of both sides, the following personal conclusions are offered :

1. The factors which influence the severity of syphilis reside both in the seed and soil, the individual and the infecting material.

2. Syphilis varies in type with the nature of the soil and the quality of the virus.

3. The nature of the soil depends upon the physical condition of the individual, his health, race, age and sex. The quality of the virus upon the dilution from frequent transmission and attenuation by mercurial treatment continuously or intermittently carried out along the links of the antecedent chain.

4. Attenuated virus and healthy soil mean abortive or benign syphilis, while with the possible exception of racial immunity or resistance, depraved soil and unmodified virus mean severe or malignant syphilis.

